# Early-Age Mechanical Properties of 3D-Printed Mortar with Spent Garnet

**DOI:** 10.3390/ma15010100

**Published:** 2021-12-23

**Authors:** Szymon Skibicki, Patrycja Jakubowska, Maria Kaszyńska, Daniel Sibera, Krzysztof Cendrowski, Marcin Hoffmann

**Affiliations:** 1 Faculty of Civil and Environmental Engineering, West Pomeranian University of Technology in Szczecin, Piastów 50a, 70-311 Szczecin, Poland; maria.kaszynska@zut.edu.pl (M.K.); daniel.sibera@zut.edu.pl (D.S.); kcendrowski@zut.edu.pl (K.C.); 2 Faculty of Mechanical Engineering and Mechatronics, West Pomeranian University of Technology in Szczecin, Piastów 19, 70-310 Szczecin, Poland; marcin.hoffmann@zut.edu.pl

**Keywords:** 3D concrete, 3D mortar, spent garnet, sustainability, fine aggregate replacement, natural sand replacement, garnet waste, aggregate replacement, recycled aggregate

## Abstract

This study determines the effect of spent garnet as a replacement for natural sand in 3D-printed mortar at early ages. Five mixes with different spent garnet amounts were prepared (0%, 25%, 50%, 75% and 100% by volume). The ratio of binder to aggregate remained unchanged. In all mixes the water/binder ratio was assumed as a constant value of 0.375. Tests were performed to confirm the printability of the mix (a path quality test using a gantry robot with an extruder). Determinations of key buildability properties of the mix (green strength and Young’s Modulus) during uniaxial compressive strength at 15 min, 30 min and 45 min after adding water were conducted. A hydraulic press and the GOM ARAMIS precision image analysis system were used to conduct the study. The results showed that an increase in spent garnet content caused a decrease in green strength and Young’s Modulus (up to 69.91% and 80.37%, respectively). It was found that to maintain proper buildability, the recommended maximum replacement rate of natural sand with garnet is 50%. This research contributes new knowledge in terms of using recycled waste in the 3D printing technology of cementitious materials.

## 1. Introduction

Issues with material properties play a key role in 3D printing technology. Evaluating the properties of a 3D printing mix often requires multiple analyses and print tests [1,2,3,4,5,6,7,8,9,10,11,12]. It should be noted that the main problem is the evaluation of the mechanical properties of fresh concrete mix [13,14,15]. There are various methods of determining these properties. Among others, Roussel et al. [16,17] and Secrieru et al. [18] tried to determine the properties of fresh mix using basic parameters (yield stress and plastic viscosity) found in rheological models. Other researchers proposed different models for determining the behavior of the mix based on classical rheological parameters (Perrot [19], Roussel [20]). Another approach is the use of methods related to soil mechanics [14,21,22,23].

Another very important factor for both printed and ordinary concretes is environmental impact [24,25,26,27,28]. The mixes used for 3D printing usually contain large amounts of binder [1,29,30,31,32]. In general, the concrete industry consumes huge amounts of sand and gravel per year. Thus, the demand for natural aggregates is increasing with the growth of the construction industry. In addition, because most research centers use only fine aggregates, these composites contain a relatively large amount of fine natural aggregate, the availability of which is becoming limited [33,34,35]. In addition, the increase in demand for natural sand not only causes its depletion, but also adversely affects the environment by causing, among others, the dredging of river beds, lowering of the water table and destruction of embankments [36,37,38]. The solution is to search for alternative aggregates [39,40]. In order to alleviate the pressure on the environment and achieve sustainable development, one of the possible solutions is to reuse waste materials for the production of construction materials. The use of waste aggregates in concretes and mortars is well known [37,41,42].

A literature review was performed based on advanced searches in Scopus and Web of Science databases (selection criteria: garnet, spent garnet, recycled sand, aggregate replacement, mortar). There are only a few studies that investigate the reuse of spent abrasives, used, e.g., for water jet cutting or for the production of concretes and mortars. In a review paper, Jamaludin et al. [36] concluded that spent garnet is a promising waste material that can be used for the production of construction materials, especially concrete. Higher hardness and finer grain size makes it suitable for use as a partial fine aggregate in concrete or brick making. Most of the research results have shown that the combination of an appropriate amount of waste garnet (25% of volume) effectively increases the strength of hardened concrete. Huseien et al. [37] focused on the reuse of garnet as a fine aggregate to replace river sand in the preparation of alkali-activated mortars. Their study showed that the workability of alkali-activated concretes increases with increasing garnet content in the cement matrix. Furthermore, replacing river sand with 25% garnet led to optimum strengths of hardened concrete and showed improvement in pore structure. The authors’ research and analysis showed that the use of available spent garnets in alkali-activated mortars is a great choice because it consumes less energy and has a lower carbon footprint and lower production costs. Muttashar et al. [38] studied the use of spent garnet to produce self-compacting concretes. A garnet replacement level of 25% showed optimum performance in both workability and mechanical properties. Increasing the proportion of garnet above 25% resulted in lower strength of self-compacting concretes. Kunchariyakun and Sukmak [43] used spent garnet as a filler in cement mortar to produce radiation-shielding concrete. The mortar containing 100% garnet showed the highest density and compressive strength, and the gamma and neutron radiation transmissions decreased as the proportion of garnet residue increased. Usman et al. [39] used garnets to obtain ordinary and self-compacting geopolymer concrete. This research showed that workability increases with the increase in garnet content. In addition, durability and strength (flexural, compressive and splitting tensile) showed improved performance with higher garnet content. However, other performance parameters were low when compared to the control mixes. Similar to previous research studies, it was shown that 25% garnet addition was the optimum percentage in the tested mortars.

In case of 3D printing of cement composites, the issue of replacing fine aggregate with waste aggregate is an under-recognized topic. Only a few studies use recycled sand for printed concretes/mortars. Based on searches in the Scopus and ScienceDirect databases (keywords: recycled sand, 3d concrete printing, 3d mortar printing), existing works on this topic were analyzed. Ding et al. [21,44] replaced up to 50% of fine aggregate with recycled sand (made by crushing and sieving demolition concrete). The grain size of the sand used in the study was up to 0.9 mm. The study showed that the higher the recycled sand content in the mix, the higher the green strength of the composite [21].

In the study [45], Ding et al. conducted studies on a fiber-reinforced concrete for 3D printing, where natural aggregate was replaced by recycled aggregate up to 50% by volume. The addition of recycled aggregate decreased the mechanical properties of the composites, especially for low amounts of fibers (0.25%). For higher amounts of fibers (1.40%) the differences were insignificant. Zhang and Xiao [46] used sand obtained by crushing waste concrete, with a particle size of 0.15 mm ÷ 2.36 mm (with fineness modulus of 2.16) for printed mortars. They substituted natural sand up to 100% by volume. The authors evaluated plastic shrinkage using an image correlation method. The results showed that mortars with a large amount of recycled sand exhibited greater plastic shrinkage. A different stud [47] uses a recycled aggregate concrete (RAC) to replace part of coarse aggregate in printable concrete (in amounts of 50% and 100%). The rheological properties and buildability were analyzed. Addition of the RAC increased the yield stress in the rheometric test. Evaluation of buildability in print tests has shown similar results for all tested composites. Mixes with the highest amounts of RAC exhibited very poor print quality (numerous surface defects and discontinuations). The above analysis shows that various research teams tried to replace, to different degrees, the natural sand used for 3D printing. The majority of research teams replaced up to 50% of the initial sand. Furthermore, there are currently no works that use residue from water jet cutting (such as spent garnet) as recycled aggregate for printed composites.

The main objective of this study is the analysis of green strength development of 3D printing mortar with spent garnet at early ages, which is a novel approach. The influence of different spent garnet replacement ratios was examined. This research proves the possibility of using spent garnet to replace natural sand in 3D-printed mortar.

## 2. Materials and Experimental Procedure

### 2.1. Materials

The key issue of this study was to analyze the possibility of using residue from abrasive wear of steel and engineering plastics, recycle it and reuse it for production of cement composites. In this regard, so-called spent garnets [36] are used. The word “garnet” refers to a group of complex silicate minerals with similar crystal structures and diverse chemical compositions [38]. Garnets have large industrial applications, which include use in waterjet cutting, production of different types of abrasives, water filtration pellets, etc. [38].

In this study, spent garnet (SG) was obtained from ORING company (Szczecin, Poland) which manufactures technical seals, mainly from rubber. The garnet was used for waterjet cutting (where high-pressure water with abrasive is used to treat materials) of construction materials. The garnet used in this work had a density of 4 g/cm^3^. The density of the garnet was determined using a helium pycnometer (Micro-Ultrapyc 1200e, Boynton Beach, FL 33426, USA).

The phase composition of the spent garnet sample was investigated using X-Ray Diffraction using Cu Kα radiation (λCu Kα = 0.1540 nm) on an Empyrean, Panalytical. Phase identification was performed using HighScore+ and the ICDD PDF-4+ 2015 database.

**Figure 1:**
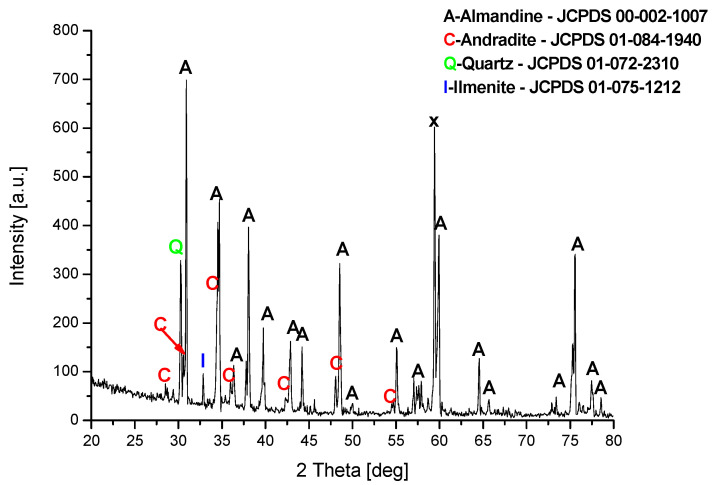
Diffraction patterns of the spent garnet.

From the documentation provided by the manufacturer, it is known that the material used for the tests consists mainly of almandine and a small amount of ilmenite, omphacite and quartz. Almandine is a mineral from the pomegranate group with the formula Al_2_Fe_3_O_12_Si_3_. Omphacite is a mineral belonging to the group of chain silicates, consisting of calcium, sodium, magnesium, iron and aluminum silicate—(Ca,Na)(Mg,Fe,Al)[Si_2_O_6_]. Ilmenite is an oxide mineral with the formula FeTiO_3_. The phase composition of the investigated material was examined using X-ray diffraction method. As shown in Figure 1, the main crystalline phase of the specimen is almandine. In addition, reflections from quartz and ilmenite as well as andradite were also observed in the tested sample. Andradite is also a mineral from the group of silicates with the formula Ca_3_Fe_2_(SiO_4_)_3_. No reflections from the omphacite were observed in the tested sample. Additionally, in Figure 1, the symbol x indicates reflections that could not be described.

The selected heavy metals for analysis of leaching to environment were copper (Cu), zinc (Zn), lead (Pb), cadmium (Cd), nickel (Ni), iron (Fe), mercury (Hg) and chromium (Cr). According to the EN-1744-3:2002 [48], SG are considered fine aggregates, and were therefore mixed with distilled water and stirred at a speed of 500 rpm for 24 h. Further, filtration was used to separate the SG from the obtained extract, using 0.45 mm sieve. Obtained extract, according to the EN-1744-3 [48], was supplemented with the 5 mL of 60% HNO_3_ on 100 mL of the solution. The extract analysis was performed using atomic absorption spectrometer (AAS) iCE 3500 (Thermo Fisher Scientific, Waltham, MA, USA). All metals, with the exception of mercury which was analyzed using cold vapors of hydrides, were analyzed using flame method. In order to prepare the calibration curve, chemicals from Spectro ECON (CHEM-LAB NV, Zedelgem, Belgium) were used. The presented results of the leaching test of SG are in Table 1. All concentrations of the heavy toxic metals in extract from the SG were below the detection limit of the spectrometer. Concentration values presented in Table 1 are simultaneously the detection limit (lowest possible measured values) of the AAS. According to the XRD analysis, the element that could be released in the greatest quantity from the SG during leaching test is iron. Iron element is present in all three major components of the garnet: Ilmenite, Almandine and Andradite. Concentration of the iron below detection limit shows that SG is a stable material in water environment. According to the EN-1744-3 [48], SG can be successfully used as an aggregate, replacing fine sand in concrete composites [49].

**Figure 2:**
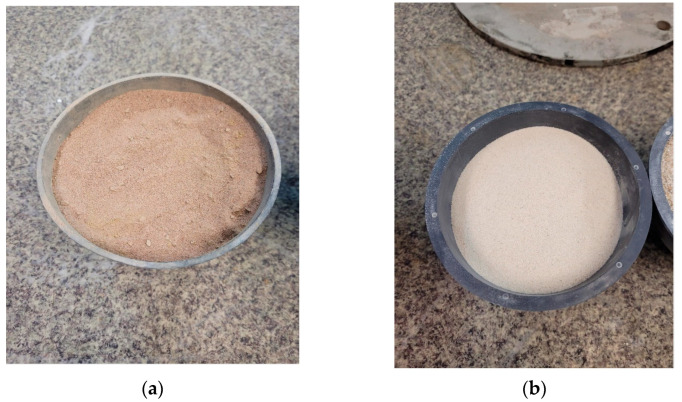
Comparison between aggregates (**a**) spent garnet (**b**) natural sand.

**Figure 3:**
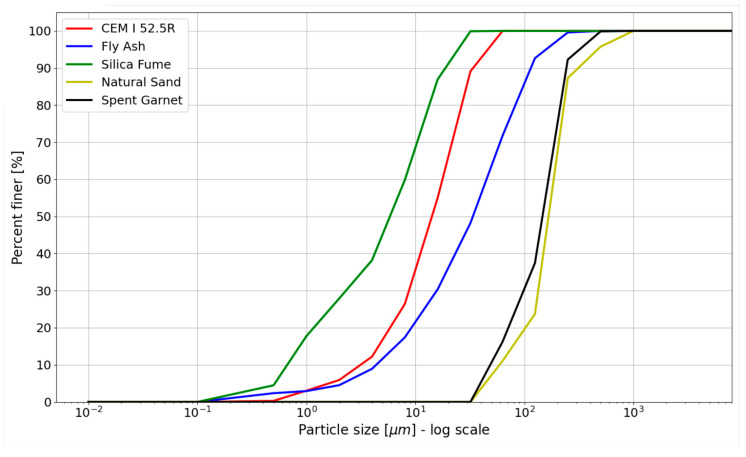
Particle size distribution of materials in the study.

Typical cementitious materials were used to prepare 3D printing mix. The mix included Portland cement CEM I 52.5 R, Coal Fly Ash from local coal power plant (Dolna Odra Power Plant, Gryfino, Poland), silica fume and natural sand (fine aggregate). The chemical compositions of cement, fly ash and silica fume are shown in Table 2. Comparison between aggregates is shown in Figure 2. Particle Size Distribution Curve for the materials used is shown in Figure 3. The curves for cement, silica fume and fly ash were obtained by laser diffraction method (in water), while those for fine aggregate and spent garnet were obtained by sieve method.

### 2.2. Mix Design

Based on an extensive literature review of cement mortars (Section 1), five mixes were designed: one control mix, OR0 (no recycled aggregate added) and four mixes with spent garnet that was used as a replacement for natural sand from 25% to 100% by volume (OR25 to OR100). In all mixes the water/binder ratio was assumed as a constant value of 0.375. Total binder amount in each mix was 1000 kg/m^3^. The binder in each mix consisted of 75% cement, 20% fly ash and 5% silica fume. The study assumes a constant slump flow of 160 ± 10 mm at 15 min after adding the water, as shown in standard [50]. This is one of the basic criteria for printed mixes (proposed in other studies [6,13,14,32,51,52]. In addition, each mix was pumped through the printing system and then printed, confirming its suitability for the task (Section 3.1). Each of the mixes was prepared by dry mixing the ingredients and adding water. The mix design is presented in Table 3. The fresh density and bulk density of the mixes are presented in Table 4.

## 3. Experimental Procedure

### 3.1. Printing Test

**Figure 4:**
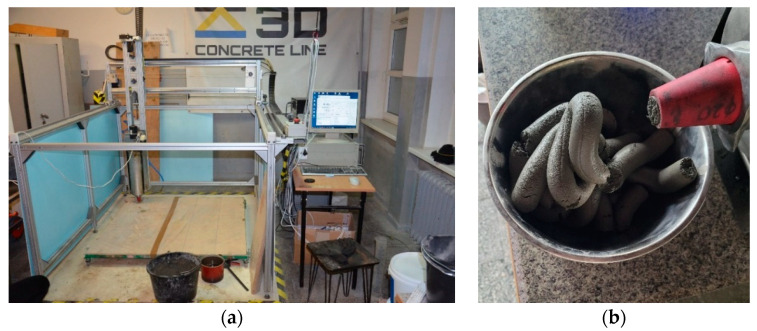
(**a**) Gantry robot with an extruder; (**b**) Mix pumped through the system.

**Figure 5:**
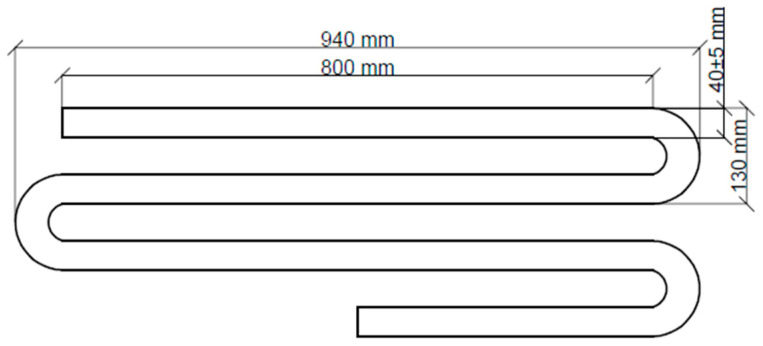
Design of the print path.

The mixes were prepared in the laboratory at 20 °C (±2°) and RH = 55% (±5%). Printing was performed using a gantry printer connected to an extruder (Figure 4a). Each mix before the printing process was initially extruded to test its suitability. The mix before green strength determination was pumped through the system (Figure 4b). To verify the printability of the designed mixes, a print quality test was performed. The test path design is shown in Figure 5. Similar tests to determine the printability of the mixes were performed by other researchers [1,29,53]. A 20 mm diameter nozzle was used for the tests. A speed of 75 mm/s was used, while the deposition rate was adjusted to extrude a path with dimensions of 40 ± 5 mm and a height of 12 ± 2 mm.

### 3.2. Test Setup and Uniaxial Compression Test

**Figure 6:**
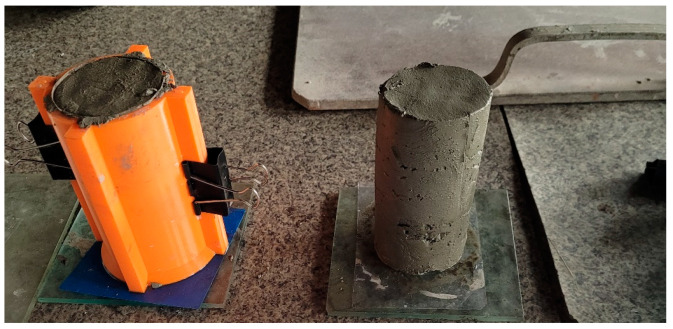
Molding of samples.

**Figure 7:**
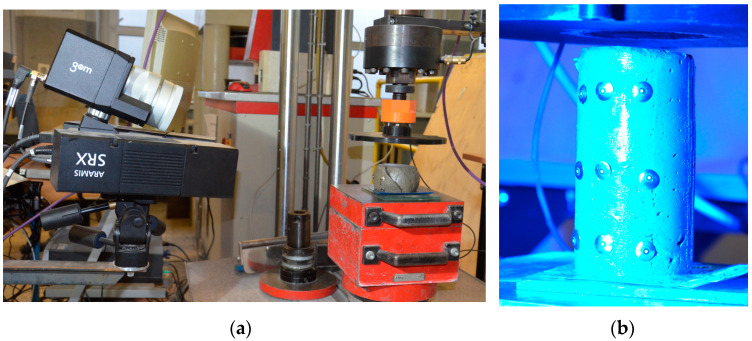
View on the test stand (**a**) ARAMIS system and sample after failure (**b**) sample at the beginning of the test.

Uniaxial unconfined compressive tests were conducted on the cylindrical specimens (D = 60 mm, H = 120 mm, Figure 6). Tests allowed for the determination of the green strength and stiffness of cement mortar. Similar approaches on green strength development were used by other authors [14,21,22,23]. The tests were performed using a hydraulic press. A loading rate of 15 mm/min was assumed for the test. In order to precisely control the results, an additional precision force sensor with a range of up to 500 N (HBM C9C 0.5 kN) coupled to a HBM MGC Plus AB22A bridge was used. Deformations during testing were recorded using the GOM ARAMIS system, which controlled the displacements of nine markers placed on the specimen (Figure 7). In addition, the HBM MGC Plus AB22A strain gauge bridge provided a real-time force signal to the ARAMIS system via an analog connector. The system configured in this way allowed for a full analysis of force–strain relationships in the GOM Correlate software (version 2020).

The test procedure was as follows: (1) dry ingredients were mixed for a minute and then water was added; (2) After two minutes of mixing, the mix was pumped through the printing system and molded, as presented in Figure 6; (3) Samples were tested at 15 min, 30 min and 45 min after molding, then approximately one minute before testing began, the sample was demolded and set up on the test bench (Figure 7b); (4) Before starting the test, nine markers were placed on the sample (Figure 7b); (5) The test was conducted until the stresses σ started to decrease (Section 4.2).

## 4. Results

### 4.1. Printing Test

**Figure 8:**
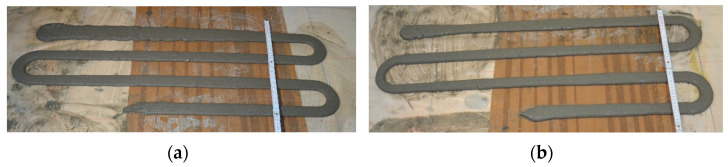
Print quality test: (**a**) OR0; (**b**) OR100.

The initial test of a print path quality was performed according to the procedure given in Section 3.1. The view of the printed path is shown in Figure 8. The dimensions of the printed paths were as given in Figure 5. Moreover, the printed paths were continuous and had no surface defects. The results showed good quality of printed paths evaluated according to other studies [1,29,53].

**Figure 9:**
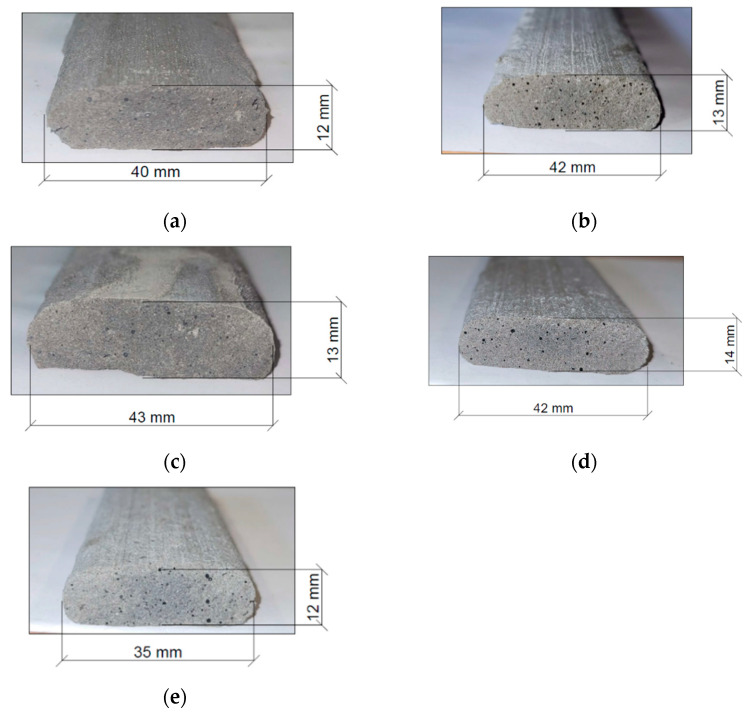
Comparison of path cross-sections: (**a**) OR0; (**b**) OR25; (**c**) OR50; (**d**) OR75; (**e**) OR100.

Figure 9 presents the cross-section of mix paths, cut after hardening. All printed mixes had the cross-section area within the assumed limits h = 12 ± 2 mm and w = 40 ± 5 mm.

All prepared mixes were positively evaluated in a print quality test.

### 4.2. Green Strength and Young’s Modulus Development

**Figure 10:**
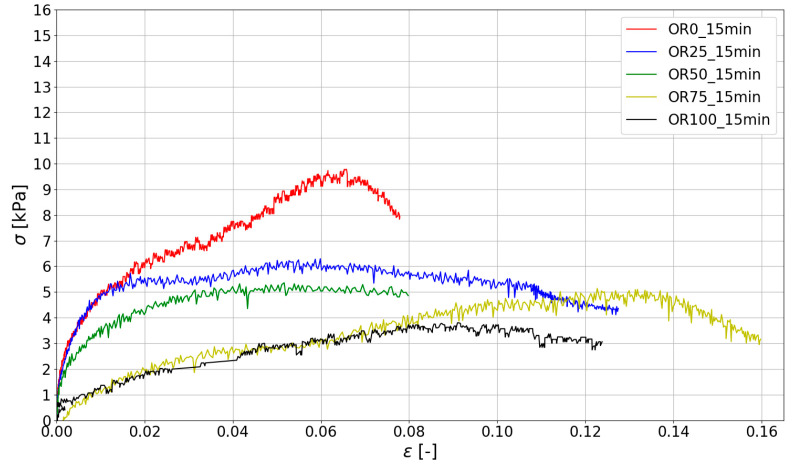
Stress–strain curves for mixes at 15 min after adding water.

**Figure 11:**
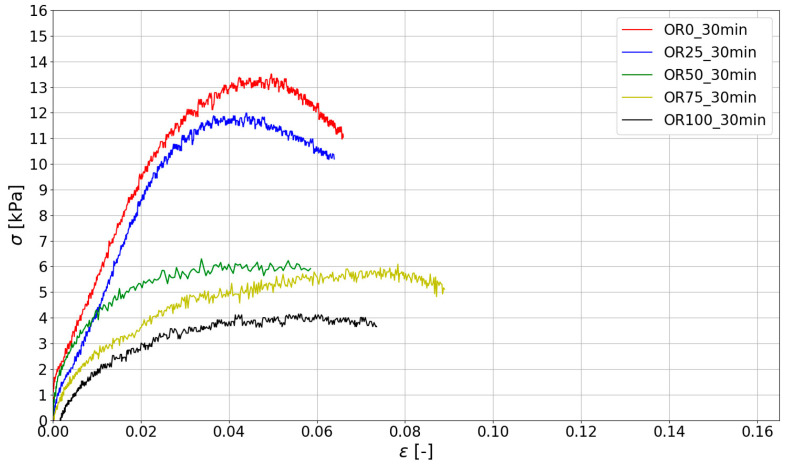
Stress–strain curves for mixes at 30 min after adding water.

**Figure 12:**
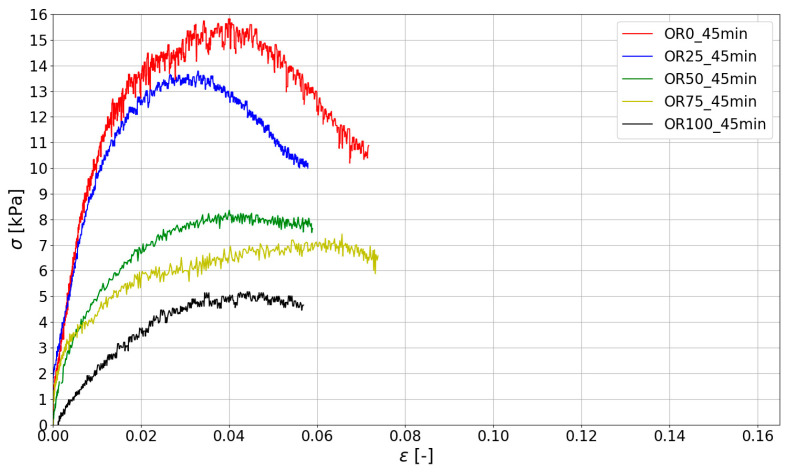
Stress–strain curves for mixes at 45 min after adding water.

Based on the measurements made with ARAMIS (Section 3.2), compressive stress–strain curves were determined for specific test times. The stresses were determined according to Equation (1). The results are shown in Figure 10, Figure 11 and Figure 12.

(1) $$\sigma \left(\epsilon \right)=\frac{N\left(\epsilon \right)}{A\left(\epsilon \right)}$$

where:

N(ϵ)—Force measured on HBM C9C 0,5kN;
A(ϵ)—Area during the test. The ARAMIS system allows to determine the real-time area, by analyzing the displacement of markers placed on the sample (in three dimensions).

The cross-sectional area of the specimen varied with time due to the change in diameter of the specimen during the test. The area was calculated on the basis of displacements of markers placed on the specimen (displacement registered in the ARAMIS system allows to locate the marker in three dimensions).

**Figure 13:**
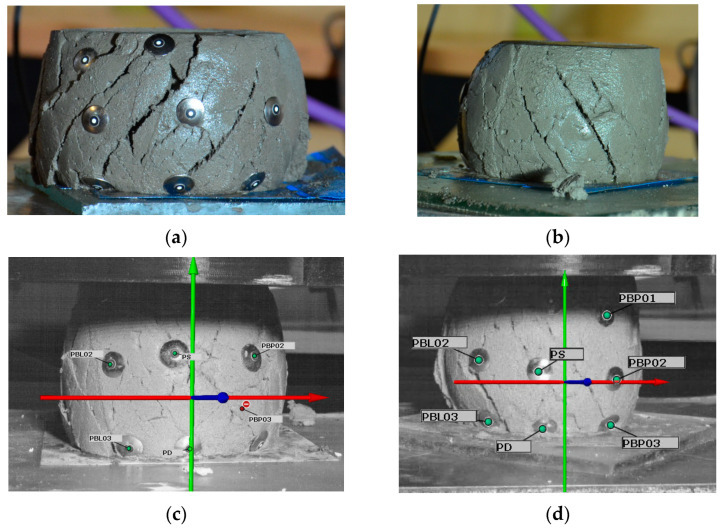
Failure mechanism of specimen: (**a**) Specimen OR50 after test; (**b**) specimen OR0 after test; (**c**) Specimen during the test—beginning of the shearing of specimen—image from the ARAMIS GOM Correlate; (**d**) Sample during the end phase of failure—image from the ARAMIS GOM Correlate.

An example of specimen failure is shown in Figure 13. All tested specimens have the same failure pattern (Figure 13a,b,d). When the axial force was increased, the cross-section of the specimen increased significantly. When the specimen reached the maximum stress σ(ϵ), the shear failure plane started to form (Figure 13c). No differences were observed between the failure mechanism for specimens with or without spent garnet. The garnet did not affect the failure pattern of the specimen regardless of the test times.

The stiffness of the fresh mix determined by Young’s Modulus plays a crucial role in 3D printing. Young’s Modulus was determined for the linear portion of the stress–strain curve σ(ϵ) up to the strain ϵ= 0.01. All the results of the green strength and Young’s Modulus are presented in Table 5, where the mean value of the maximum stress ($\sigma_{max}$) and Young’s Modulus ($E_{0.01}$) in the study are given. For all obtained data, the values of coefficient of variation (CoV) were calculated.

Table 5 presents a percentile reduction of the values in relation to the control sample without spent garnet, calculated as (2):

(2) $$X_{red}=\frac{X_{OR0}-X_{ORX}}{X_{OR0}}\cdot 100\%$$

where:

$X_{red}$—percentage reduction of value ($\sigma_{max,red}$—for maximum stresses; $E_{0.01,red}$—for Young’s Modulus);
$X_{OR0}$—mean value for the control specimen;
$X_{ORX}$—mean value for specimen with spent garnet.

**Figure 14:**
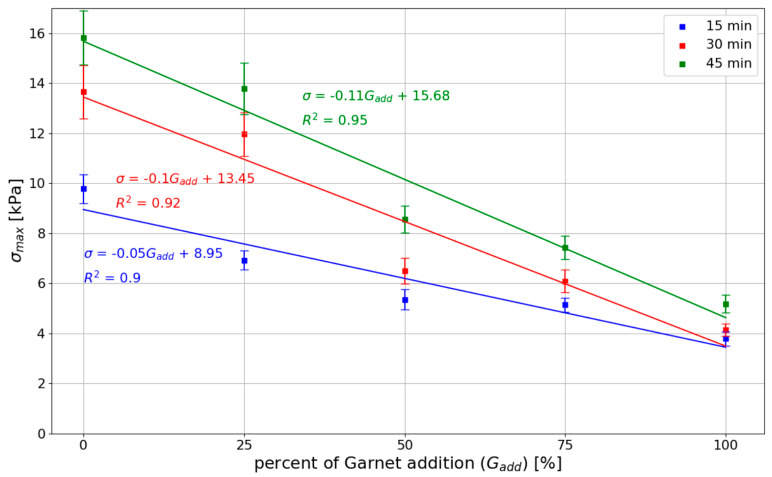
Relationship between garnet content and maximum green strength.

Analyzing the data presented in Table 4, it should be noted that the highest strength and stiffness were reported for mixes with natural sand (without spent garnet). The reduction in $\sigma_{max}$ is almost directly proportional to the amount of garnet content (a visible linear relationship between the spent garnet content and the maximum green strength). Figure 14 shows the analysis of the test results using the linear regression method. The data were divided into three groups according to the time of testing (15 min, 30 min, 45 min). It can be concluded that the decrease in green strength is linear for all tested specimens. The values of R^2^ for all considered sets of data are above 0.9, which represents a very good fit. A decrease in strength of about 50% (45.20–52.40%) was observable for a replacement rate of 50%. For mixes with a 100% replacement rate, a decrease in $\sigma_{max}$ of between 61.16% and 67.21% was observed.

**Figure 15:**
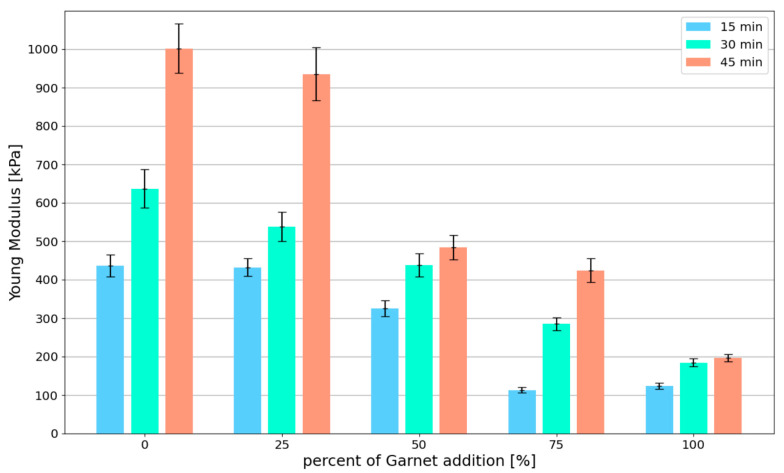
Relationship between the stiffness and garnet content.

Figure 15 shows the relationship between stiffness (Young Modulus—$E_{0.01})$ and garnet content. When the changes in stiffness of fresh mix are analyzed, it can be concluded that $E_{0.01}$ also decreases with the increase in SG content. For specimens tested after 15 min and 30 min, the decrease in $E_{0.01}$ is linear and the maximum reduction in green strength is between 70% and 80%. In addition, it should be noted that for a test time of t = 45 min, the stiffness decrease is significantly more observable for higher SG content (50%, 75% and 100%).

## 5. Discussion

Results obtained in this study concur with results found elsewhere. Huseien et al. [37] showed that garnet increases the workability of mixes (reduces $\tau_{0}$). According to [37], this phenomenon is caused by the water demand of the garnet. Similar results were presented by Usman et al. [39]; the total water absorption by garnet is lower, which increases the workability of the mix. Muttashar et al. [38] showed that the workability of the mix increased by 29% when 100% of natural aggregate was replaced with garnet. The authors concluded that this is caused by the different grain shapes of garnet and natural aggregate. Other study [38] indicated that the porous nature of spent garnet contributes the most to the increased workability. The team of Kunchariyakun et al. [43] showed that for a 100% replacement ratio, the dry density of the specimen increased by about 39% and water absorption decreased by about 56% compared to the control sample. This influenced the workability of the mix. The researchers proposed that this is due to the relatively low water absorption of garnet and the dense structure of the cement mortar. In conclusion with respect to traditional mortars, it can be seen that increasing the amount of spent garnet improves the workability of the mix, resulting in a reduction of its yield stress ($\tau_{0}$). This in turn reduces the green strength of 3D-printed mortars.

Table 6 shows the results of green strength and Young’s Modulus of 3D-printed mixes obtained in different studies. The only study that utilized recycled sand was conducted by Ding et al. [21]. In their study, the sand was obtained by recycling demolition waste. The maximum stresses obtained in this study were between 13.65–15.82 kPa. Those results were slightly higher than those obtained by Ding et al. [21] (9.51–10.68 kPa) for the control mix without recycled sand. Results presented in Ding et al. [21] showed that with the increase in the replacement ratio, the maximum stresses also increased. Even though the general trend is different (in comparison to this study), the results for the replacement rates of 25% and 50% are similar between the studies (this study $\sigma_{max}=6.30\;–13.78\;kPa$) to those obtained by Ding et al. [21] ($\sigma_{max}=8.89-12.23\;kPa$).

In this study, the mixes without garnet achieved maximum stresses of 9.78 kPa and 15.82 kPa, which are in agreement with other studies, including Casagrande et al. [14] $\sigma_{max}=3.82\;kPa-\sigma_{max}=26.04\;kPa$; Wolfs et al. [22] $\sigma_{max}=7.71\;kPa-\sigma_{max}=10.05\;kPa$; and Panda et al. [23] $\sigma_{max}=10.65\;kPa$.

This study has shown that replacing at least 50% of natural sand with garnet significantly reduces the mechanical properties of fresh mortar. The maximum stresses achieved for the mentioned replacement ratio barely reach the minimum values found elsewhere in the literature. For the garnet replacement rate of 75% and above, the $\sigma_{max}\;$obtained in this study is below most of the results presented in the literature ($\sigma_{max}\leq 7.43\;kPa$). This means that in the context of maintaining proper buildability, the recommended maximum replacement rate of garnet is 50%.

Results obtained in this study do not significantly deviate from the studies found in the literature [14,21,22,23]. However, determinations of the green properties of mortars with recycled spent garnet are a novelty in this field.

## 6. Conclusions

This paper examines the effect of spent garnet as a replacement for natural sand in 3D-printed mortar at early ages. Tests were carried out on five printing mixes with different sand replacement rates (0%, 25%, 50%, 75% and 100%). Uniaxial compressive strength tests were performed at 15 min, 30 min and 45 min after adding water, in order to determine green strength and Young’s Modulus. The tests were performed using a hydraulic press and the GOM ARAMIS precision image analysis system, which recorded the deformation of the specimen in real time. According to the test results and analysis, the following conclusions can be drawn:

- The maximum stresses obtained in this study in the uniaxial compressive test for the control mix are higher or equal to the results obtained by other researchers [14,21,22,23]. In addition, print quality tests (Section 4.1) were performed for all tested mixes. The mixes designed in this study meet the requirements for printing mixes and have sufficient buildability.
- Replacing the natural sand with spent garnet resulted in a decrease in maximum stress and Young’s Modulus. Significant decreases in green strength and Young’s Modulus were achieved for replacement rates of 75% and 100% (a decrease in $\sigma_{max}$ up to 69.61% compared to control mix; a decrease in $E_{0.01}$ up to 80.37%). Results for the mentioned replacement ratio obtained in this study were lower than the ones found in the in the literature [14,21,22,23] ($\sigma_{max}\leq 7.43\;kPa$). This means that in the context of maintaining proper buildability, the recommended maximum replacement rate of natural sand with garnet is 50%. A detailed analysis is presented in Section 5.
- All specimens have a similar failure pattern (Section 4.2). As the axial force was increased, the cross-section of the specimen increased significantly. When the specimen reached the maximum stress σ(ϵ), shear failure plane formation began. No differences were observed between the failure mechanisms for specimens with or without spent garnet. The content of the spent garnet did not affect the failure pattern regardless of test time.

## Figures and Tables

**Table 1:** Metals’ leaching concentrations from the spent garnet.

Element	Concentration [mg/L]	Method of Metal Analysis
Zn	0.011	Flame—air/acetylene
Cd	0.016	Flame—air/acetylene
Cu	0.037	Flame—air/acetylene
Ni	0.053	Flame—air/acetylene
Pb	0.074	Flame—air/acetylene
Fe	0.053	Flame—air/acetylene
Hg	0.0008	Hydrides—cold vapors
Cr	0.011	Flame—nitrous oxide/acetylene

**Table 2:** Chemical composition of Portland cement, fly ash and silica fume.

Chemical Composition, % by Weight	CEM I 52.5 R	Fly Ash	Silica Fume
SiO_2_	19.70	54.00	94.00
Al_2_O_3_	4.93	28.40	–
Fe_2_O_3_	2.54	7.30	–
CaO	64.23	3.10	0.30
CaCO_3_	–	–	–
MgO	1.32	2.40	–
SO_3_	2.91	0.40	1.90
Na_2_O	0.12	1.10	–
K_2_O	0.76	2.90	–
Cl^-^	0.07	0.01	0.10
H_2_O	–	–	0.70
Na_2_0eq	0.63	–	–
LOI	–	–	3.00

**Table 3:** Mix design.

Mix	Spent Garnet [% by Vol]	Natural Sand [% by Vol]	Cement CEM I 52.5R [kg]	Fly Ash [kg]	Silica Fume [kg]
OR0	0	0	750	200	50
OR25	25	75			
OR50	50	50			
OR75	75	25			
OR100	100	0			

**Table 4:** Density of studied mixes.

Mix	Fresh Density [kg/m^3^]	Bulk Denity [kg/m^3^]
OR0	2053.6	1908.69
OR25	2133.5	1960.41
OR50	2255.1	2111.57
OR75	2313.6	2175.23
OR100	2409.1	2348.03

**Table 5:** Comparison of the maximum compressive stresses (green strength, $\sigma_{max}$) and Young’s Modulus ($E_{0.01}$).

Recycled Sand Replacement Ratio	Rest Time [min]	$\sigma_{max}$ [kPa]	CoV [%]	$\sigma_{max,red}$ [%]	$E_{0.01}$ [kPa]	CoV [%]	$E_{0.01,red}$ [%]
0	15	9.78	5.9%	-	436.32	6.6%	-
	30	13.65	7.8%	-	636.82	7.9%	-
	45	15.82	6.9%	-	1002.26	6.5%	-
25	15	6.92	5.5%	−29.26%	432.50	5.4%	−0.88%
	30	11.96	7.3%	−12.39%	538.11	7.0%	−15.50%
	45	13.78	7.5%	−12.87%	935.21	7.4%	−6.69%
50	15	5.36	7.4%	−45.20%	325.59	6.2%	−25.38%
	30	6.50	8.0%	−52.40%	437.56	6.9%	−31.29%
	45	8.56	6.3%	−45.89%	483.92	6.4%	−51.72%
75	15	5.14	5.5%	−47.45%	113.30	6.1%	−74.03%
	30	6.09	7.5%	−55.38%	285.61	5.8%	−55.15%
	45	7.43	6.4%	−53.03%	424.42	7.2%	−57.65%
100	15	3.80	7.4%	−61.16%	124.05	6.2%	−71.57%
	30	4.15	5.8%	−69.61%	184.86	5.9%	−70.97%
	45	5.19	6.9%	−67.21%	196.78	5.0%	−80.37%

**Table 6:** Evaluation of green strength in different studies.

Research Team	Recycled Sand Replacement [% by Vol]	Rest Time t [min]	Maximum Stress [kPa]	Young Modulus [kPa]	Specimen [mm]
Ding et al. [21]	0	15	-	-	75 × 150
	0	30	9.51	28.82	
	0	45	10.68	31.9	
	25	15	-	-	
	25	30	8.89	32.9	
	25	45	11.41	29.9	
	50	15	-	-	
	50	30	9.06	27.92	
	50	45	12.23	36.47	
Casagrande et al. [14]	-	15	3.82–12.54	94–256	60 × 120
	-	30	4.30–26.04	93–488	
	-	45	-	-	
Wolfs et al. [22]	-	15	7.71	99	70 × 140
	-	30	10.05	117	
	-	45	-	-	
Panda et al. [23]	-	15	-	-	70 × 140
	-	30	10.65	350	
	-	45	-	-	

## Data Availability

The data presented in this study are available upon reasonable request from the corresponding author.
